# Low-Level Environmental Cadmium Exposure Is Associated with DNA Hypomethylation in Argentinean Women

**DOI:** 10.1289/ehp.1104600

**Published:** 2012-03-01

**Authors:** Mohammad Bakhtiar Hossain, Marie Vahter, Gabriela Concha, Karin Broberg

**Affiliations:** 1 Department of Laboratory Medicine, Division of Occupational and Environmental Medicine, Lund University, Lund, Sweden; 2 International Centre for Diarrhoeal Disease Research, Bangladesh, Dhaka, Bangladesh; 3 Institute of Environmental Medicine, Karolinska Institutet, Stockholm, Sweden; 4 Risk and Benefit Assessment Department, National Food Agency, Uppsala, Sweden

**Keywords:** cadmium, *DNMT1*, *DNMT3B*, epigenetic, genotype, *LINE-1*, *MLH1*, *p16*, pyrosequencing

## Abstract

Background: Cadmium, a common food pollutant, alters DNA methylation *in vitro*. Epigenetic effects might therefore partly explain cadmium’s toxicity, including its carcinogenicity; however, human data on epigenetic effects are lacking.

Objective: We evaluated the effects of dietary cadmium exposure on DNA methylation, considering other environmental exposures, genetic predisposition, and gene expression.

Methods: Concentrations of cadmium, arsenic, selenium, and zinc in blood and urine of nonsmoking women (*n* = 202) from the northern Argentinean Andes were measured by inductively coupled mass spectrometry. Methylation in CpG islands of *LINE-1* (long interspersed nuclear element-1; a proxy for global DNA methylation) and promoter regions of *p16* [cyclin-dependent kinase inhibitor 2A (*CDKN2A*)] and *MLH1* (mutL homolog 1) in peripheral blood were measured by bisulfite polymerase chain reaction pyrosequencing. Genotyping (*n* = 172) for the DNA (cytosine-5-)-methyltransferase 1 gene (*DNMT1* rs10854076 and rs2228611) and DNA (cytosine-5-)-methyltransferase 3 beta gene (*DNMT3B* rs2424913 and rs2424932) was performed with Sequenom iPLEX GOLD SNP genotyping; and gene expression (*n* = 90), with DirectHyb HumanHT-12 (version 3.0).

Results: Cadmium exposure was low: median concentrations in blood and urine were 0.36 and 0.23 µg/L, respectively. Urinary cadmium (natural log transformed) was inversely associated with *LINE-1* methylation (β = –0.50, *p* = 0.0070; β = –0.44, *p* = 0.026, adjusted for age and coca chewing) but not with *p16* or *MLH1* methylation. Both *DNMT1* rs10854076 and *DNMT1* rs2228611 polymorphisms modified associations between urinary cadmium and *LINE-1* (*p*-values for interaction in adjusted models were 0.045 and 0.064, respectively). The rare genotypes demonstrated stronger hypomethylation with increasing urinary cadmium concentrations. Cadmium was inversely associated with *DNMT3B* (*r*_S_ = –0.28, *p* = 0.0086) but not with *DNMT1* expression (*r*_S_ = –0.075, *p* = 0.48).

Conclusion: Environmental cadmium exposure was associated with DNA hypomethylation in peripheral blood, and *DNMT1* genotypes modified this association. The role of epigenetic modifications in cadmium-associated diseases needs clarification.

Cadmium is a widespread environmental pollutant associated with adverse health effects, including cancer, kidney damage, low bone-mineral density, bone fractures, and probably impaired early-life development (Engström A et al. 2011; Järup and Åkesson 2009; Kippler et al. 2011). The general population is exposed to cadmium mainly through the diet, in particular, through cereals and vegetables, and through smoking. Potential mechanisms of cadmium toxicity described so far include interactions with sulfhydryl groups and endocrine disruption, as well as oxidative stress (Hartwig 2010; Johnson et al. 2003; Monroe and Halvorsen 2006; Xu et al. 2011; Yu et al. 2010).

DNA methylation is essential for embryogenesis and for maintaining cell-lineage–specific gene expression throughout life. Dysregulation of epigenetic processes, such as DNA methylation, may lead to impaired childhood development or chronic diseases later in life, including cancer (Faulk and Dolinoy 2011; Feinberg 2007; Robins et al. 2011). In carcinogenesis, global DNA methylation is generally decreased, whereas specific regions of DNA that contain tumor suppressor genes often show hypermethylation (Chalitchagorn et al. 2004; Deng et al. 2006; Gaudet et al. 2003; Robertson and Jones 2000). *In vitro* studies have indicated that cadmium may interfere with epigenetic processes: Both gene-specific DNA hypermethylation with gene silencing of *p16INK4a* [*CDKN2A* (cyclin-dependent kinase inhibitor 2A)], *RASSF1* [Ras association (RalGDS/AF-6) domain family member 1], and *MT1* (metallothionein 1) genes, and global DNA hypomethylation have been reported (Benbrahim-Tallaa et al. 2007; Huang et al. 2008; Jiang et al. 2008; Martinez-Zamudio and Ha 2011; Takiguchi et al. 2003). However, there are no reports on the epigenetic effects of cadmium exposure in humans.

The aim of this study was to evaluate whether environmental cadmium exposure, mainly through the normal diet, may affect DNA methylation, both of repetitive genetic elements (as a proxy for global methylation) and of specific genes.

## Materials and Methods

*Participants.* We studied 202 women living in the Andean plateau (3,800 m above sea level) in northern Argentina, an area that has minimal industrial or vehicle-derived pollution. Most of the study participants (161) were from the village of San Antonio de los Cobres, which has about 5,000 inhabitants. The 41 remaining participants were from small surrounding villages. The study area is known to have elevated concentrations of arsenic (Vahter et al. 1995), but not cadmium (Concha et al. 2010), in the drinking water.

The study individuals were recruited with the assistance of local medical personnel, except in the small mining village of Tolar Grande, where we went from house to house to explain the study and invited the adults to participate. We included only women because men were often away from home for longer periods for work and were therefore likely to be exposed to a different variety of environmental hazards. Furthermore, women may be more susceptible to cadmium exposure than are men because of their increased gastrointestinal cadmium absorption when iron stores are low (Berglund et al. 1994), as is common in women of childbearing age. Only four study participants smoked cigarettes, and one reported drinking alcohol, but almost half (46.5%) reported that they chewed coca leaves. As reported before, interviews with the study participants revealed that almost all women drank exclusively public water and that their diets consisted mainly of corn, beans, chicken, and pork (Broberg et al. 2011). Only three women reported taking any medication at the time of the study: one being treated for gastritis and two for hypertension. No women reported any history of or current malignancy.

We measured two biomarkers of cadmium exposure: blood cadmium, reflecting ongoing exposure, and urinary cadmium, reflecting long-term exposure (Järup and Åkesson 2009). Both oral and written informed consent was provided by all the study participants. The study was approved by the Ministry of Health in Salta, Argentina, and the Regional Ethical committee at Karolinska Institute, Sweden.

*Blood and urine collection.* All biological samples were nonfasting spot samples collected during the daytime at local health clinics or the hospital in San Antonio de los Cobres. Peripheral blood for DNA extraction (*n* = 202) was collected in K_2_EDTA tubes (Vacuette®; Vacuette Bio One, Frickenhausen, Greiner, Germany), blood for element analysis (*n* = 202) in lithium heparin tubes (Vacuette Bio One), and blood for RNA extraction (*n* = 122) in PAXgene Blood RNA Tubes (Becton Dickinson, Franklin Lakes, NJ, USA). Spot urine samples were collected and processed as described previously (Concha et al. 1998). Blood, plasma, and urine samples were kept at –20°C before and after the transport (with cooling blocks) to Sweden for analysis.

*Analysis of elements in blood and urine.* We measured cadmium in blood and urine, and selenium and zinc in blood, because these elements may influence DNA methylation or one-carbon metabolism (Hong et al. 2000; Pilsner et al. 2011; Smeester et al. 2011). Cadmium, selenium, and zinc were measured using inductively coupled plasma mass spectrometry (ICP-MS; model 7500ce; Agilent Technologies, Waldbronn, Germany) with a collision/reaction cell system to remove potential interferences, particularly interference from molybdenum in the cadmium analysis (Concha et al. 2010; Kippler et al. 2009). The limit of detection (LOD; 3 × SD of blank values) was 0.001 µg/L for urinary cadmium, and no sample had a cadmium concentration below the LOD. For blood measurements, the LOD was 0.011, 0.081, and 0.49 µg/L for cadmium, selenium, and zinc, respectively, and no samples were below the LOD for any of the compounds measured. Analytical accuracy was controlled by analyzing commercially available reference materials (Concha et al. 2010; Kippler et al. 2009). Because the study area is known to have varying concentrations of arsenic in the drinking water, and because arsenic may alter DNA methylation (Chanda et al. 2006; Smeester et al. 2011), we also assessed exposure to arsenic by measuring arsenic metabolites (inorganic arsenic, methylarsonic acid, and dimethylarsinic acid) in urine using HPLC/ICP-MS (Engström et al. 2010). Additionally, the study area was recently found to have elevated concentrations of boron, lithium, and cesium in the drinking water, and we measured these elements in urine by ICP-MS (Concha et al. 2010). To control for variation in urine dilution, we adjusted the cadmium and arsenic concentrations in urine to the mean specific gravity of urine (1.020 g/mL) (Nermell et al. 2008). We measured the specific gravity of the urine samples with a digital refractometer (model RD 712 clinical refractometer; EUROMEX, Arnhem, Holland).

*Polymerase chain reaction (PCR) and pyrosequencing.* DNA was isolated using the QIAamp® DNA Blood Midi kit (Qiagen, Hilden, Germany). We used the EpiTect® kit (Qiagen) to treat 1 μg of DNA (50 ng/μL) with bisulfite. Bisulfite treatment of DNA converts unmethylated cytosine residues into uracil, leaving the methylated cytosine unchanged; these changes can be quantified by pyrosequencing. The bisufite-treated DNA was stored at –20°C until used for PCR.

We used the bisulfite-treated DNA (0.6–1 μL) in a 15- to 25-μL PCR reaction using the Pyromark PCR kit (Qiagen). One of the PCR primers was biotinylated, and the PCR product was purified using Streptavidin Sepharose High Performance beads (Amersham Biosciences, Uppsala, Sweden). The Sepharose beads with the immobilized PCR products were purified, washed, and denatured with 0.2 M NaOH and washed again using a vacuum prep tool (Pyrosequencing Inc., Westborough, MA, USA). After annealing 12 µL of the 0.3-µmol/L pyrosequencing primer to the purified single-stranded PCR product, we performed pyrosequencing using the PSQ HS96 Pyrosequencing System (Qiagen). The degree of methylation was expressed as the percentage of methylated cytosines over the sum of methylated and unmethylated cytosines. We verified bisulfite conversion using non-CpG (cytosine–guanine dinucleotide) cytosine residues as built-in controls, and complete conversion of cytosine at a non-CpG site indicated successful bisulfite conversion.

LINE-1, p16, *and* MLH1 *methylation.* As a surrogate measure of genome-wide methylation, we measured peripheral blood DNA methylation in the long interspersed nuclear element-1 (*LINE-1*) retrotransposable elements that make up about 17% of human DNA (Lander et al. 2001). We also measured methylation of CpG islands in *p16* [cyclin-dependent kinase inhibitor 2A (*CDKN2A*)], a tumor suppressor gene that is often hypermethylated in tumors and may be relevant in cadmium-induced tumorigenesis (Benbrahim-Tallaa et al. 2007), and in the *MLH1* (mutL homolog 1) gene, a component of the DNA mismatch repair pathway.

Commercially available kits (Qiagen) were used to measure the methylation of four CpG sites in *LINE-1* [positions 305–331 of GenBank accession no. X58075 (National Library of Medicine 2012a)], seven CpG sites in *p16* (+148 to +174 in exon 1; GenBank accession no. L27211), and four CpG sites in *MLH1* (–209 to –188; GenBank accession no. U07418) following the manufacturer’s instructions. A single PCR fragment spanning a part of each genetic element was amplified, and the degree of methylation was analyzed in a single pyrosequencing reaction using 20 µL PCR product for *LINE-1*, 3 µL for *p16*, and 4 µL for *MLH1*. The samples were analyzed as singlets for *LINE-1* and as triplicates for *p16* and *MLH1*. We selected 10% of the samples at random and reassayed them. The repeatability of the method, expressed as the variation in coefficients, was 2.0%, 36.7%, and 26.4%, for *LINE-1*, *p16*, and *MLH1* methylation, respectively. Previous reports have validated pyrosequencing as an effective technique for measuring *LINE-1* methylation at the population level (Schernhammer et al. 2010; Tarantini et al. 2009).

*Genotyping and gene expression.* Genotyping for polymorphisms of DNA methyltransferase-1 [(cytosine-5-)-methyltransferase 1] (*DNMT1* rs10854076 and rs2228611, one from each linkage disequilibrium block in the gene) and DNA (cytosine-5-)-methyltransferase 3 beta (*DNMT3B* rs2424913 and rs2424932) was performed on DNA from peripheral blood with iPLEX GOLD SNP genotyping (Sequenom Inc., San Diego, CA, USA) the Swegene DNA facility at Malmö University Hospital (Malmö, Sweden), as described previously (Engström K et al. 2011). *DNMT1* rs2228611 [minor allele frequency (MAF) = 40%] is a synonymous amino acid exchange, whereas rs10854076 (MAF = 35%) is situated in an intron. *DNMT3B* rs2424913 (MAF = 5%) and rs2424932 (MAF = 6%) are both situated in introns. The functional impact of these polymorphisms on DNA methylation, if any, is unknown.

RNA was extracted using the PAXgene Blood RNA kit (PreAnalytiX, Hombrechtikon, Switzerland) and stored at –80°C until isolation. We evaluated RNA concentration and purity using a NanoDrop spectrophotometer (NANODROP 1000; Thermo Scientific, Wilmington, DE, USA), and we evaluated the RNA integrity number (RIN) using a Bioanalyzer 2100 (Agilent Technologies, Santa Clara, CA, USA). The results indicated good RNA quality (RIN > 7.5) for all of the samples. Of 122 available PAX tubes for RNA extraction, 90 samples were selected for gene expression analysis based on their RNA quality and the amount of RNA left for analysis [for descriptive characteristics of this subset of the study population, see Supplemental Material, Table 1 (http://dx.doi.org/10.1289/ehp.​1104600)]. Of the 90 individuals selected, 75 were not first-degree relatives, and they were selected for the analysis of genetic effect modification on the association between cadmium exposure and epigenetic markers. DirectHyb HumanHT-12 (version 3.0; Illumina, San Diego, CA, USA) was used for gene expression analysis according to the manufacturer’s instructions at the SCIBLU (Swegene Centre for Integrative Biology at Lund University) facility at Lund University. Background signals were filtered from the gene expression data by BioArray Software Environment (BASE) (Vallon-Christersson et al. 2009). The DirectHyb HumanHT-12 (HT-12) array (Illumina) included one *DNMT1* transcript and five *DNMT3B* transcripts [the gene has alternatively spliced transcripts (National Library of Medicine 2012b)]. No *LINE-1* transcripts were included on the array.

**Table 1:** Descriptive information of the women and biomarkers analyzed.^a^

Variable	Median	5th–95th percentile
Age (years)		34		18–64
Body weight (kg)		57.0		44.0–79.9
Height (cm)		152		144–161
BMI (kg/m2)		24.7		18.8–35.0
Blood hemoglobin (g/L)		155		130–180
Urinary creatinine (g/L)b		0.90		0.52–1.4
Coca chewing (%)		46.5		
Methylation (%)				
LINE1c		86.2		83.5–89.3
p16d		3.4		1.7–5.8
MLH1e		4.2		1.2–7.1
Blood measures				
Cadmium (μg/L)		0.36		0.20–0.76
Selenium (μg/L)		176		150–221
Zinc (mg/L)		6.8		5.5–8.2
Urinary measures				
Cadmium (μg/L)b		0.23		0.091–0.70
Arsenic (μg/L)b		230		21–545
Boron (mg/L)b		14.6		2.6–27.9
Cesium (μg/L)b		470		26.1–880
Lithium (mg/L)b		3.9		0.27–10.4
an = 202 subjects, except for creatinine (n = 194) and blood selenium (n = 201) analyses. bAdjusted for specific gravity of urine (1.020 g/mL). cAverage of relative amounts of methylated cytosines in four CpGs, positions 305–331, GenBank accession no. X58075. dAverage of relative amounts of methylated cytosines in seven CpGs, positions 148–174, accession no. L27211. eAverage of relative amounts of methylated cytosines in four CpGs, positions –209 to –188, accession no. U07418.				

*Statistical analyses.* We used the averages of the relative amounts of methylated cytosine residues in the four, seven, and four CpG sites measured in *LINE-1*, *p16*, and *MLH1*, respectively to determine the overall methylation level for each genetic element analyzed in a given sample. We used Spearman’s rank correlation analysis to estimate univariate correlations between cadmium in blood or urine and methylation and gene expression, as well as correlations with potential covariates such as age, body weight, body mass index (BMI), use of coca leaves, and zinc and selenium concentrations in whole blood.

We first evaluated the gene methylation by quintiles of cadmium exposure [see Supplemental Material, Figures 1–3 (http://dx.doi.org/10.1289/ehp.1104600)] and by scatter plots. Because visual inspection of the plots suggested linear relationships, we used linear regression models to estimate associations (β-coefficients) of cadmium in blood and urine with the epigenetic markers. We performed a multivariable-adjusted analysis controlling first for age and coca chewing (yes/no) because age and coca chewing were correlated with at least one of the epigenetic markers (*p*-values < 0.2) and because cadmium exposure–outcome coefficients changed by > 5% when they were included in models. To assess the robustness of associations, we additionally controlled for urinary arsenic, boron, lithium, and cesium (modeled as untransformed continuous variables). In the linear regression analyses, urine and blood cadmium concentrations were natural log (ln) transformed to obtain normal distributions of the residuals and a better linear fit with the outcomes.

The influence of *DNMT1* genotypes (effect modification) on the association between urinary cadmium concentration and *LINE-1* methylation was analyzed in individuals that were not first-degree relatives (*n* = 172). This analysis was performed by modeling a cross-product term between genotype (for *DNMT1* as an ordinal variable with three categories, and for *DNMT3B* dichotomized because few individuals were variant homozygotes) and lower-order terms for genotype and ln-urinary cadmium concentrations. Analyses were then stratified by genotype to estimate associations between urinary cadmium and *LINE-1* methylation according to each genotype. In addition, we used stratified models to estimate associations between urinary cadmium and *DNMT1* expression according to genotype in the 75 participants that were not first-degree relatives. Associations between genotypes and the expression of DNMTs were estimated using analysis of variance with and without adjustment for age and coca chewing. We did not adjust *p*-values for multiple comparisons. A *p*-value < 0.05 was considered statistically significant. All statistical analyses were performed using SPSS (version 18; SPSS, Chicago, IL, USA).

## Results

Descriptive data for the study participants and element concentrations in blood and urine are presented in Table 1. In general, the women were relatively short, and, using the conventional cut-offs for overweight (BMI 25–29.9) and obesity (BMI > 30), about half of them were overweight and 18% obese. Blood concentrations of hemoglobin, selenium, and zinc indicated adequate nutritional status. Cadmium concentrations were 0.013–1.5 µg/L in urine (median, 0.23 µg/L) and 0.17–1.1 µg/L in blood (median, 0.36 µg/L). Four women who reported smoking had cadmium concentrations of 0.045–0.24 µg/L in urine and 0.18–0.52 µg/L in blood. Median *LINE-1*, *p16*, and *MLH1* methylation levels were 86.2%, 3.4%, and 4.2%, respectively (Table 1).

The epigenetic markers were positively correlated with each other: the correlation between *p16* and *MLH1* methylation being the strongest (*r*_S_ = 0.78, *p* < 0.01), followed by *LINE-1* and *MLH1* (*r*_S_ = 0.51, *p* < 0.01), and *LINE-1* and *p16* (*r*_S_ = 0.42, *p* < 0.01). Urinary cadmium concentrations were negatively correlated with *LINE-1* methylation (*r*_S_ = –0.19, *p* < 0.01). Blood cadmium concentrations were not correlated with *LINE-1* (*r*_S_ = 0.046) but were positively correlated with urinary cadmium concentration (*r*_S_ = 0.46, *p* < 0.01). Neither urinary cadmium nor blood cadmium concentrations were correlated with *p16* or *MLH1* methylation (*r*_S_ < 0.1). None of the nutritional markers (BMI, hemoglobin, selenium, and zinc) were significantly correlated with the epigenetic markers (*r*_S_ < 0.11).

In the linear regression analysis, the ln-transformed urinary cadmium concentrations of all women (*n* = 202) were inversely associated with *LINE-1* methylation [for unadjusted model: β = –0.50; 95% confidence interval (CI): –0.86, –0.14; *p* = 0.0070; Table 2]. Estimates did not change much after adjustments for age and coca chewing or after additional adjustment for urinary arsenic concentration (Table 2). Further adjustments for urinary boron, lithium, or cesium also had little influence on effect estimates (data not shown). When the analysis for *LINE-1* included an interaction term between cadmium and dichotomized arsenic in urine (median split), no significant interaction was detected (*p* = 0.37). Excluding the four smokers (which left *n* = 198) did not change the estimated association between urinary cadmium and *LINE* methylation (in fully adjusted model: β = –0.44; 95% CI: –0.85, –0.034). There was no association between ln-transformed urinary or blood cadmium concentrations and *p16* or *MLH1* methylation (Table 2).

**Table 2:** Associations between ln-transformed blood and urinary cadmium concentration (μg/L) and percent methylation of LINE1, p16, and MLH1.

LINE1		p16		MLH1	
Predictor	β (95% CI)	p-Value	β (95% CI)	p-Value	β (95% CI)	p-Value
Blood cadmium									
Unadjusteda		0.16 (–0.46, 0.78)	0.61		0.11 (–0.38, 0.60)	0.65		0.14 (–0.52, 0.80)	0.69
Adjusted 1b		0.40 (–0.27, 1.07)	0.24		0.30 (–0.23, 0.83)	0.26		0.26 (–0.46, 0.98)	0.47
Adjusted 2c		0.45 (–0.23, 1.12)	0.19		0.24 (–0.29, 0.77)	0.37		0.19 (–0.53, 0.91)	0.61
Urinary cadmium									
Unadjusteda		–0.50 (–0.86, –0.14)	0.0070		–0.13 (–0.42, 0.15)	0.37		–0.062 (–0.45, 0.33)	0.75
Adjusted 1b		–0.44 (–0.83, –0.053)	0.026		–0.048 (–0.36, 0.26)	0.76		–0.0054 (–0.43, 0.42)	0.98
Adjusted 2c		–0.42 (–0.82, –0.025)	0.038		–0.11 (–0.42, 0.21)	0.51		–0.073 (–0.50, 0.36)	0.74
β is the unstandardized regression coefficient. aUnivariate analysis (n = 202): percent methylation = α1 + β1 cadmium in blood/urine. bAdjusted analysis (n = 202): percent methylation = α1 + β1 cadmium in blood/urine + β2 × age + β3 × coca chewing. cAdjusted analysis (n = 201): percent methylation = α1 + β1 cadmium in blood/urine + β2 × age + β3 × coca chewing + β4 arsenic in urine.									

The association of urinary cadmium with *LINE-1* methylation showed statistically significant interactions in unadjusted models with the genotypes *DNMT1* rs10854076 and rs2228611 (Table 3). The association between urinary cadmium and *LINE-1* methylation was significantly stronger in carriers of the CC and CG genotypes of *DNMT1* rs10854076 than in common GG carriers, and in carriers of the rare GG *DNMT1* rs2228611 genotype than in GA and AA carriers. *DNMT3B* genotypes did not significantly modify the association between urinary cadmium and *LINE-1* methylation, although associations were stronger among women with the variant genotypes of both *DNMT3B* polymorphisms.

**Table 3:** Effect modification of DNMT1 and DNMT3B genotypes on the association between ln-transformed cadmium concentrations in urine and for LINE1 methylation levels (n = 172 in all analyses).

Unadjusted analysis			Adjusted analysis		
Genotype	β^a^ (95% CI)	p-Value^b^	β^c^ (95% CI)	p-Value^d^
DNMT1								
rs10854076								
CC (n = 25)		–1.09 (–2.02, –0.15)		0.041		–0.99 (–2.03, 0.059)		0.045
CG (n = 69)		–0.96 (–1.68, –0.24)				–1.04 (–1.91, –0.17)		
GG (n = 78)		–0.052 (–0.58, 0.47)				–0.053 (–0.61, 0.51)		
rs2228611								
GG (n = 35)		–1.19 (–1.99, –0.40)		0.050		–1.04 (–1.96, –0.12)		0.064
GA (n = 68)		–0.38 (–1.07, 0.31)				–0.38 (–1.14, 0.38)		
AA (n = 69)		–0.10 (–0.67, 0.47)				–0.13 (–0.75, 0.48)		
DNMT3B								
rs2424913								
CC + CT (n = 15)		–1.22 (–3.20, 0.77)		0.39		–1.04 (–3.36, 1.27)		0.38
TT (n = 155)		–0.45 (–0.84, –0.064)				–0.39 (–0.81, 0.024)		
rs2424932								
AA + GA (n = 18)		–1.36 (–3.12, 0.40)		0.28		–1.43 (–3.39, 0.53)		0.25
GG (n = 152)		–0.46 (–0.85, –0.065)				–0.39 (–0.81, 0.037)		
aPercent LINE1 methylation (stratified for each genotype) = α1 + β1 cadmium. bp-Value interaction from term β3 in the model: percent LINE1 methylation = α1 + β1 genotype + β2 cadmium + β3 (genotype × cadmium). cPercent LINE1 methylation (stratified for each genotype) = α1 + β1 cadmium + β2 age + β3 coca chewing. dp-Value interaction from term β3 in the model: percent LINE1 methylation = α1 + β1 genotype + β2 cadmium + β3 (genotype × cadmium) + β4 age + β5 coca chewing.								

Characteristics of the 90 women included in the analyses of DNMT gene expression [see Supplemental Material, Table 1 (http://dx.doi.org/10.1289/ehp.1104600)] were similar to those of all participants (Table 1), except that the subset was slightly younger (median, 32 vs. 34 years; *p* = 0.019). *DNMT1* expression in blood was much higher than *DNMT3B* expression (Table 4). *DNMT3B* _1733929 [Illumina HT-12 transcripts are named ILMN_(number)] expression was significantly higher in *DNMT1* rs10854076 CC and CG carriers than in GG carriers, and in *DNMT3B* rs2424913 TT carriers than in rare C allele carriers (Table 4). Urinary cadmium concentration was inversely correlated with expression of one *DNMT3B* transcript (ILMN_1733929; *r*_S_ = –0.28, *p* = 0.0086) but not with the other four transcripts (*r*_S_ = –0.12 to 0.20; *p* = 0.062–0.78) or with *DNMT1* expression (*r*_S_ = –0.075, *p* = 0.48). Urinary cadmium also was not associated with *DNMT1* expression when stratified by *DNMT1* rs10854076 genotype (CC + CG carriers: β = –19; 95% CI: –88, 50; GG carriers: β = 6.0; 95% CI: –63, 75) or by *DNMT1* rs2228611 genotype (GG + GA carriers: β = –17.6; 95% CI: –80, 45; AA allele carriers: β = –0.60; 95% CI: –75, 74). There were no correlations between *DNMT1* or *DNMT3B* (ILMN_1733929) expression and *LINE-1* methylation (*r*_S_ = 0.050 and –0.16, respectively; *p*-values = 0.64 and 0.14).

**Table 4:** DNMT1 and DNMT3B gene expression according to DNMT1 and DNMT3B genotypes.^a^

DNMT1					DNMT3B				
Genotype	Gene expressionb	p-Valuec	p-Valued	Gene expressionb	p-Valuec	p-Valued
DNMT1												
rs10854076												
CC (n = 8)		654.9		0.14		0.18		92.2		0.027		0.040
CG (n = 30)		640.3						90.0				
GG (n = 37)		629.6						87.3				
rs2228611												
GG (n = 12)		653.6		0.18		0.18		91.0		0.23		0.26
GA (n = 31)		639.6						89.2				
AA (n = 32)		591.4						87.8				
DNMT3B												
rs2424913												
CC + CT (n = 7)		583.0		0.83		0.67		85.1		0.068		0.036
TT (n = 67)		640.0						89.2				
rs2424932												
AA + GA (n = 8)		559.0		0.68		0.38		86.4		0.19		0.11
GG (n = 66)		640.3						89.1				
aDNMT1 is represented by one transcript (ILMN_1760201), whereas DNMT3B is represented by five transcripts (values from ILMN_1733929 presented here). DNMT genotypes were not associated with the expression of other DNMT3B transcripts (p > 0.1). bMedian, expressed as an arbitrary unit. cp-Value from model gene expression = α1 + β1 × genotype. dp-Value from model gene expression = α1 + β1 × genotype + β2 × age + β3 × coca chewing.												

## Discussion

We found that low-level urinary cadmium in women, which represents life-long cadmium exposure, was inversely associated with *LINE-1* methylation, a marker of global DNA methylation. Urinary cadmium exposure was also inversely associated with the expression of *DNMT3B*, which is involved in *de novo* CpG methylation, but not with expression of *DNMT1*, which is primarily involved in maintaining CpG methylation patterns in the genome. The inverse association between urinary cadmium and *LINE-1* methylation varied according to *DNMT1* polymorphisms; the inverse association was stronger among carriers of the rare alleles of *DNMT1* rs10854076 and rs2228611. The gene expression data from blood show associations between polymorphisms *DNMT1* rs10854076 and *DNMT3B* rs2424913 and *DNMT3B* gene expression.

Most likely, diet was the main source of cadmium for the study subjects. Only a few of the women smoked cigarettes, another major source of cadmium exposure, and judging from their blood cadmium concentrations (0.18–0.52 µg/L), it can be assumed they did not smoke much. Furthermore, the study area has minimal industrial pollution and little vehicle traffic. The urine cadmium concentrations in our study population (median, 0.23 µg/L; range, 0.013–1.5 µg/L; or a median of 0.26 µg/g creatinine) were similar to those reported for women of similar age in the United States (median, 0.19 µg/g creatinine) (Gallagher et al. 2011). A slightly higher concentration has been reported for an Asian population (median, 0.59 µg/L), which is possibly due to dietary exposure through rice, which easily takes up cadmium from soil (Kippler et al. 2007). Thus, if the observed association between cadmium and hypomethylation reflects a causal relation, effects may be common and stronger in groups with higher exposures, such as smokers and occupationally exposed workers. However, further studies in other populations, including occupationally exposed individuals, are warranted.

CpG sites in *LINE-1* promoters are usually heavily methylated, and genome-wide methylation loss from these sites has been regarded as a common epigenetic event in malignancies that may play a role in carcinogenesis (Cho et al. 2010; Choi et al. 2009; Wilhelm et al. 2010). There is increasing concern that cadmium acts as a metalloestrogen (Fechner et al. 2011) and that it might increase the risk of hormone-related cancers in humans (Åkesson et al. 2008; Gallagher et al. 2010; Siewit et al. 2010). The association found in the present study between cadmium and *LINE-1* hypomethylation in women suggests that cadmium can alter DNA methylation, which could be another mechanism for cadmium carcinogenesis. *In vitro* data lend some support to this hypothesis: Takiguchi et al. (2003) reported that cadmium exposure of nontransformed rat cells resulted in the inhibition of methyltransferase activity and, at higher exposures, hypomethylation. They also showed that after 10 weeks of exposure the cells became malignantly transformed and had increased methyltransferase activity. This phenomenon has also been shown in malignantly transformed prostate epithelial cells: cadmium exposure increased DNMT enzymatic activity and overexpression of *DNMT3B* accompanied by hypermethylation (Benbrahim-Tallaa et al. 2007).

Little is known about the potential effects of environmental pollutants on *LINE-1* methylation. In a recent U.S. bladder cancer case–control study, higher toenail concentrations of arsenic (> 75th percentile) were associated with reduced *LINE-1* methylation (Wilhelm et al. 2010). In contrast, urinary arsenic was not associated with *LINE-1* in the present study. Possibly, this can be explained by specific genetic polymorphisms in the *AS3MT* [arsenic (+3 oxidation state) methyltransferase] gene that previously were found in the present study population to be associated with a very efficient methylation and detoxification of inorganic arsenic (Engström K et al. 2011). Other factors may also influence *LINE-1* methylation. Exposure to coarse particulate matter (particulate matter with aerodynamic diameter ≤ 10 µm) (Tarantini et al. 2009) and black carbon (Baccarelli et al. 2009) were found to be inversely associated with *LINE-1* methylation in peripheral blood cells from humans. Maternal prenatal smoking was associated with lower *LINE-1* methylation in buccal cells of preschool children with glutathione *S*-transferase mu 1 (*GSTM1*)-null genotype, whereas there was higher methylation in the *GSTM1*-present children (Breton et al. 2009).

The maintenance of methylation in somatic cells is largely carried out by *DNMT1* (Chen and Li 2006), and *DNMT1* expression in normal peripheral blood has previously been reported to be higher than *DNMT3B* expression (Mizuno et al. 2001), consistent with our findings. DNMT1 and DNMT3B colocalize in the nucleus (Kim et al. 2002), and it has been shown that in cancer cells DNMT1 and DNMT3B work cooperatively to maintain DNA methylation (Rhee et al. 2002). Of note, the allelic frequencies of *DNMT3B* single-nucleotide polymorphisms were low in the present study, limiting the possibility to detect modest gene–environment interactions. Further, because the levels of *DNMT3B* expression were lower than those of *DNMT1*, these findings should be interpreted cautiously.

There was no association between blood cadmium concentrations, which represent a measure of short-term exposure, and our measure of global DNA methylation, although cadmium levels in blood and urine were moderately correlated. If a causal relation exists between cadmium exposure and DNA methylation, one may speculate that the cadmium effect is chronic in nature or occurred a long time before the analyses, even early in life. Furthermore, we found no effect of blood hemoglobin, selenium, or zinc status on the epigenetic markers. The lack of association between selenium concentrations and global DNA methylation in our study population is in contrast to the results of Pilsner et al. (2011), who measured plasma selenium in relation to global leukocyte DNA methylation. The difference in results may reflect differences in selenium status; the women in the present study all had adequate blood selenium concentrations (138–250 µg/L), whereas the Bangladeshi individuals studied by Pilsner et al. (2011) showed plasma concentrations of 45–149 µg/L (plasma concentrations are somewhat lower than selenium in whole blood), indicating lower selenium status. Other differences include the methods used to measure global DNA methylation: Pilsner et al. (2011) used a [^3^H]-methyl incorporation assay assessing methylation at all genomic CpG sites, whereas we used bisulfite-PCR pyrosequencing to quantitate *LINE-1* methylation at four CpG sites, which served as a surrogate for global DNA methylation.

We conducted this study on a fairly homogeneous population in which subjects differed very little in terms of diet, air pollution, and other lifestyle factors, including smoking and alcohol consumption. This homogeneity may explain the small variation in degree of *LINE-1* methylation we found compared with other studies (Choi et al. 2009; Tarantini et al. 2009). Also, we adjusted for other essential and toxic elements, such as arsenic, but these elements did not appear to modify the association between cadmium and *LINE-1* methylation. A limitation of our study is that we measured methylation in the DNA isolated from peripheral blood only and not from individual cell populations or other tissues. DNA methylation patterns may differ among blood cell types (Wu et al. 2011).

## Conclusion

We found that low-level environmental cadmium exposure was associated with a marker of global DNA hypomethylation in peripheral blood. This association was modified in relation to *DNMT1* genotypes. Future studies are necessary to confirm our findings in other populations and determine whether cadmium-associated epigenetic modification is present in other tissues, and if epigenetic effects of cadmium may play a role in cadmium-associated diseases.

## Supplemental Material

(45 KB) PDF
