# Supplementary material for: Low-Level Environmental Cadmium Exposure Is Associated with DNA Hypomethylation in Argentinean Women
Source: Environ Health Perspect. 2012 Mar 1;120(6):879–84. doi: 10.1289/ehp.1104600 (PMC3385444; doi:10.1289/ehp.1104600)
Supplement: (45 KB) PDF [file ehp.1104600.s001.pdf]

Supplemental Material

Low-Level Environmental Cadmium Exposure is Associated with DNA Hypomethylation in Argentinean Women

Mohammad Bakhtiar Hossain[1,2], Marie Vahter[3], Gabriela Concha[4], Karin Broberg[1*]

[1]Department of Laboratory Medicine, Division of Occupational and Environmental Medicine, Lund University, Lund, SE-22185 Sweden

[2]International Centre for Diarrhoeal Disease Research, Bangladesh (ICDDR,B), Dhaka, Bangladesh

[3]Institute of Environmental Medicine, Karolinska Institutet, Stockholm, Sweden

[4]National Food Agency, Risk and Benefit Assessment Department, 751 26 Uppsala, Sweden.

[*] Correspondence: Karin Broberg, Department of Laboratory Medicine, Division of Occupational and Environmental Medicine, Lund University. Klinikgatan 21, SE-22185 Lund, Sweden. Telephone: +46 (0) 46 173819. Fax: +46 (0) 46 143207. E-mail: karin.broberg_palmgren@med.lu.se.

Table of Contents

Supplemental Material, Table 1. Descriptive information and biomarker concentrations in blood and urine of women (N=90) analyzed for gene expression 3

Supplemental Material, Figure 1. Mean fraction of methylated *LINE1* in peripheral lymphocytes according to quintiles of cadmium concentration in urine 5

Supplemental Material, Figure 2. Mean fraction of methylated *p16* in peripheral lymphocytes according to quintiles of cadmium concentration in urine 6

Supplemental Material, Figure 3. Mean fraction of methylated *MLH1* in peripheral lymphocytes according to quintiles of cadmium concentration in urine 7

Supplemental Material, Table 1. Descriptive information and biomarker concentrations in blood and urine of women (N=90) analyzed for gene expression[a]

Variables	Median	5 - 95 percentiles
Age (years)	32.0	15.0 – 60.5
Body weight (Kg)	55.5	41.3 – 77.5
Height (cm)	153	145 - 163
BMI (kg/m^2)	24.0	18.0 – 33.1
Blood hemoglobin (g/L)	153	118 - 181
Urinary creatinine (g/L)[b]	0.90	0.56-1.3
Coca chewing (%)	43.3	
LINE1 methylation (%)[c]	85.7	83.6 - 89.1
p16 methylation (%)[d]	3.2	1.7 - 5.8
MLH1 methylation (%)[e]	3.6	1.1 - 7.1
Blood cadmium (µg/L)	0.32	0.19 - 0.64
Urinary cadmium (µg/L)[b]	0.20	0.092 - 0.61
Urinary arsenic (µg/L)[b]	204	15.6 - 622
Blood selenium (µg/L)	178	154 - 220
Blood zinc (mg/L)	6.8	5.3 - 8.2
Urinary boron (mg/L)[b]	13.1	2.0 – 25.8
Urinary cesium (µg/L)[b]	412	14.0 – 1152
Urinary lithium (mg/L)[b]	3.2	0.17 – 10.4

[a]Information available from N=90 subjects, except for urinary creatinine (N=86).

[b]Adjusted for specific gravity of urine (1.020 g/ml).

[c]Average of relative amounts of methylated Cs in 4 CpGs +305-331, GenBank accession X58075.

[d]Average of relative amounts of methylated Cs in 7 CpGs +148-174, acc. L27211.

[e]Average of relative amounts of methylated Cs in 4 CpGs -209- -188, acc. U07418.

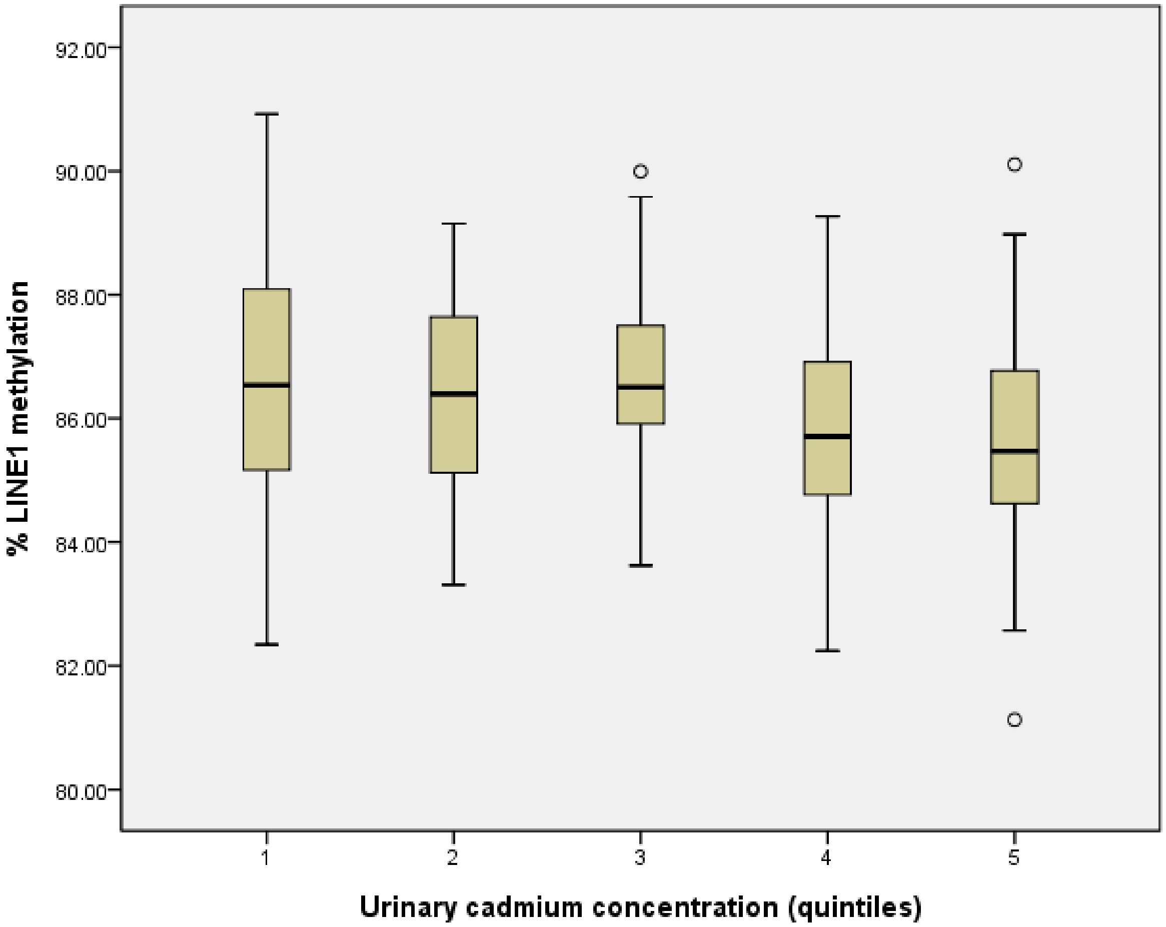

Supplemental Material, Figure 1. **Mean fraction of methylated *LINE1* in peripheral lymphocytes according to quintiles of cadmium concentration in urine.** Boxes extend from the 25th to the 75th percentile, horizontal bars represent the median, whiskers represent the range of the values, and outliers (values more than 1.5 box-lengths away from the box) are represented as points. Urinary cadmium concentration quintile 1: 0.013 to ≤0.129, quintile 2: >0.129 to ≤0.185, quintile 3: >0.185 to ≤0.265, quintile 4: >0.265 to ≤0.373, and quintile 5: >0.373 to ≤1.52 µg/L.

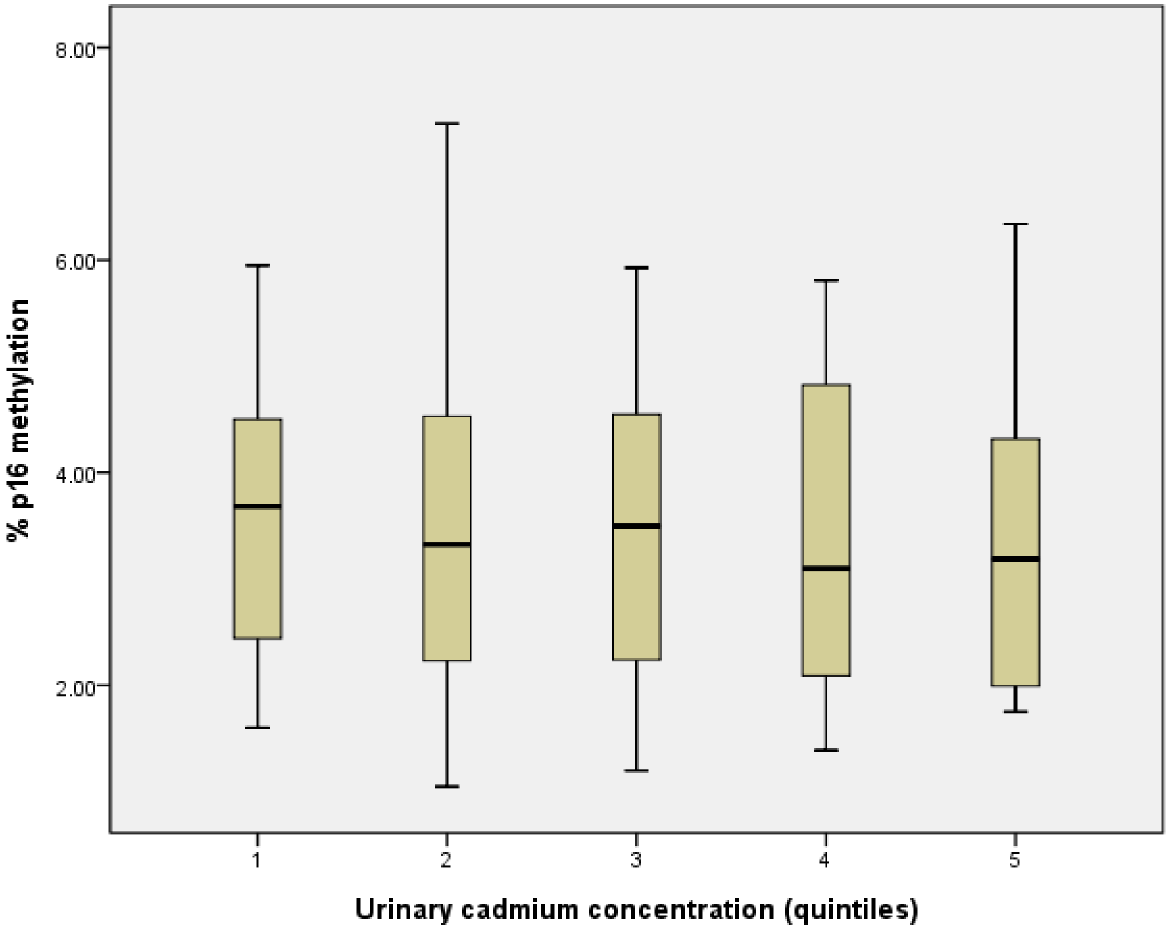

Supplemental Material, Figure 2. **Mean fraction of methylated *p16* in peripheral lymphocytes according to quintiles of cadmium concentration in urine.** Boxes extend from the 25th to the 75th percentile, horizontal bars represent the median, and whiskers represent the range of the values. Urinary cadmium concentration quintile 1: 0.013 to ≤0.129, quintile 2: >0.129 to ≤0.185, quintile 3: >0.185 to ≤0.265, quintile 4: >0.265 to ≤0.373, and quintile 5: >0.373 to ≤1.52 µg/L.

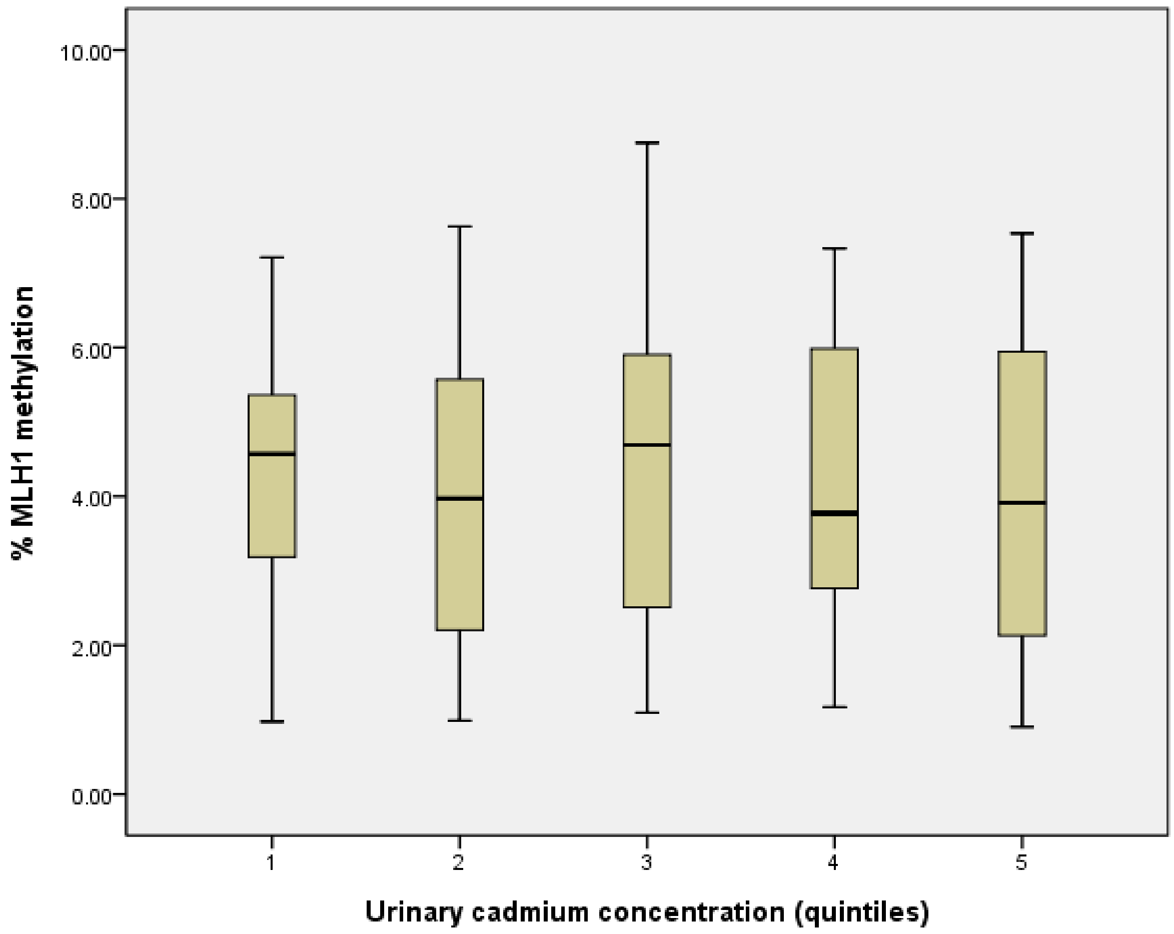

Supplemental Material, Figure 3. **Mean fraction of methylated *MLH1* in peripheral lymphocytes according to quintiles of cadmium concentration in urine.** Boxes extend from the 25th to the 75th percentile, horizontal bars represent the median, and whiskers represent the range of the values. Urinary cadmium concentration quintile 1: 0.013 to ≤0.129, quintile 2: >0.129 to ≤0.185, quintile 3: >0.185 to ≤0.265, quintile 4: >0.265 to ≤0.373, and quintile 5: >0.373 to ≤1.52 µg/L.
